# Supplementary material for: The Brief Health Literacy Scale for Adults: Adaptation and Validation of the Health Literacy for School-Aged Children Questionnaire
Source: Int J Environ Res Public Health. 2023 Nov 16;20(22):7071. doi: 10.3390/ijerph20227071 (PMC10671482; doi:10.3390/ijerph20227071)
Supplement: Supplementary file 1 [file ijerph-20-07071-s001.zip › ijerph-2649065-supplementary.pdf]

International Journal of Environmental Research and Public Health

MDPI

Supplementary Materials

The Brief Health Literacy Scale for Adults: Adaptation and Validation of the Health Literacy for School-Aged Children Questionnaire

Stinne Eika Rasmussen [1,2,*], Anna Aaby [2], Anne Søjbjerg [1,2], Anna Mygind [1], Helle Terkildsen Maindal [2], Olli Paakkari [3] and Kaj Sparle Christensen [1,2]

[1] Research Unit for General Practice, Bartholins Allé 2, 8000 Aarhus C, Denmark; annsoj@ph.au.dk (A.S.); mygind@ph.au.dk (A.M.); kasc@ph.au.dk (K.S.C.)
[2] Department of Public Health, Aarhus University, Bartholins Allé 2, 8000 Aarhus C, Denmark; annaab@rm.dk (A.A.); htm@ph.au.dk (H.T.M.)
[3] Faculty of Sport and Health Sciences, Research Centre for Health Promotion, University of Jyväskylä, Keskussairaalantie 4, 40014 Jyväskylä, Finland; olli.paakkari@jyu.fi
* Correspondence: stinne.e.rasmussen@ph.au.dk

Table S1. Overview of the wording of the original Health Literacy for School-Aged Children (HLSAC) instrument in English [1], the Danish version of the HLSAC [2], the English version of the Brief Health Literacy scale for Adults (B-HLA), and the Danish version of the B-HLA.

	**HLSAC original English version**	**HLSAC Danish version**	**B-HLA English version**	**B-HLA Danish version**
Instructing text	From the following options, choose the one that best describes your opinion.<br>I am confident that…	Vælg den mulighed nedenfor, der beskriver din mening bedst (Kun ét kryds ved hvert spørgsmål). Jeg er sikker på at…	From the following options, choose the one that best describes your opinion.<br>I am confident that…	Vælg den mulighed nedenfor, der bedst beskriver din mening.<br>Jeg er sikker på at…
Response categories	Not at all true<br>Not completely true<br>Somewhat true<br>Absolutely true	Slet ikke rigtigt<br>Ikke helt rigtigt<br>Nogenlunde rigtigt<br>Helt rigtigt	Not at all true<br>Not completely true<br>Somewhat true<br>Absolutely true	Slet ikke rigtigt<br>Ikke helt rigtigt<br>Nogenlunde rigtigt<br>Helt rigtigt
Items				
1	I have good information about health	Jeg har god viden om sundhed	I have good information about health	Jeg har god viden om sundhed
2	When necessary, I am able to give ideas on how to improve health in my immediate surroundings (e.g., a nearby place or area, family, friends)	Hvis der er brug for det, kan jeg komme med ideer til at forbedre sundhed i mine nære omgivelser (fx et sted i nærheden, min familie, venner)	I am able to give ideas on how to improve health in my immediate surroundings (e.g., a nearby place or area, family, friends)	Jeg kan komme med ideer til at forbedre sundheden i mine nære omgivelser (fx et sted i nærheden, min familie, venner)
3	I can compare health-related information from different sources	Jeg kan sammenligne information om sundhed fra forskellige kilder	I can compare health-related information from different sources	Jeg kan sammenligne information om sundhed fra forskellige kilder
**4**	I can follow the instructions given to me	Jeg kan følge de instruktioner, jeg får fra	I can follow the advice given to me by	Jeg kan følge de anvisninger, jeg får fra

	by healthcare personnel (e.g., nurse, doctor)	sundhedspersonale (fx sygeplejerske eller læge)	healthcare personnel (e.g., nurse, doctor)	sundhedspersonale (fx sygeplejerske eller læge)
**5**	I can easily give examples of things that promote health	Jeg kan nemt komme med eksempler på noget, der forbedrer sundhed	I can easily give examples of things that promote health	Jeg kan nemt komme med eksempler på noget, der forbedrer sundhed
**6**	I can judge how my own actions affect the surrounding natural environment	Jeg kan vurdere, hvordan mine handlinger påvirker miljøet omkring mig	I can judge how my own actions affect the surrounding natural environment	Jeg kan vurdere, hvordan mine handlinger påvirker naturen omkring mig
**7**	When necessary I find health-related information that is easy for me to understand	Hvis jeg har brug for det, finder jeg information om sundhed, som jeg let kan forstå	I can find health-related information that is easy for me to understand	Jeg kan finde information om sundhed, som jeg let kan forstå
**8**	I can judge how my behaviour affects my health	Jeg kan vurdere, hvordan min adfærd påvirker min sundhed	I can judge how my behaviour affects my health	Jeg kan vurdere, hvordan min adfærd påvirker min sundhed
**9**	I can usually figure out if some health-related information is right or wrong	Jeg kan som regel finde ud af, om information om sundhed er rigtigt eller forkert	I can usually decide if some health-related information is right or wrong	Jeg kan som regel vurdere, om information om sundhed er rigtig eller forkert
**10**	I can give reasons for choices I make regarding my health	Jeg kan forklare de valg jeg træffer i forhold til min sundhed	I can provide reason for choices I make regarding my health	Jeg kan begrunde de valg jeg træffer i forhold til min sundhed

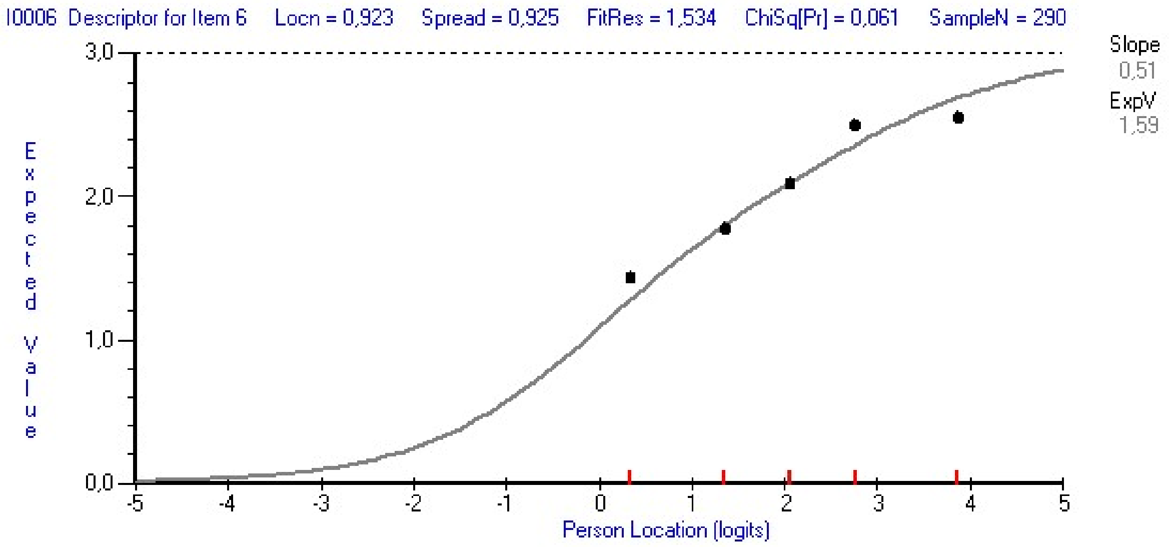

Figure S1. Item Characteristic Curve (ICC) for item 6.

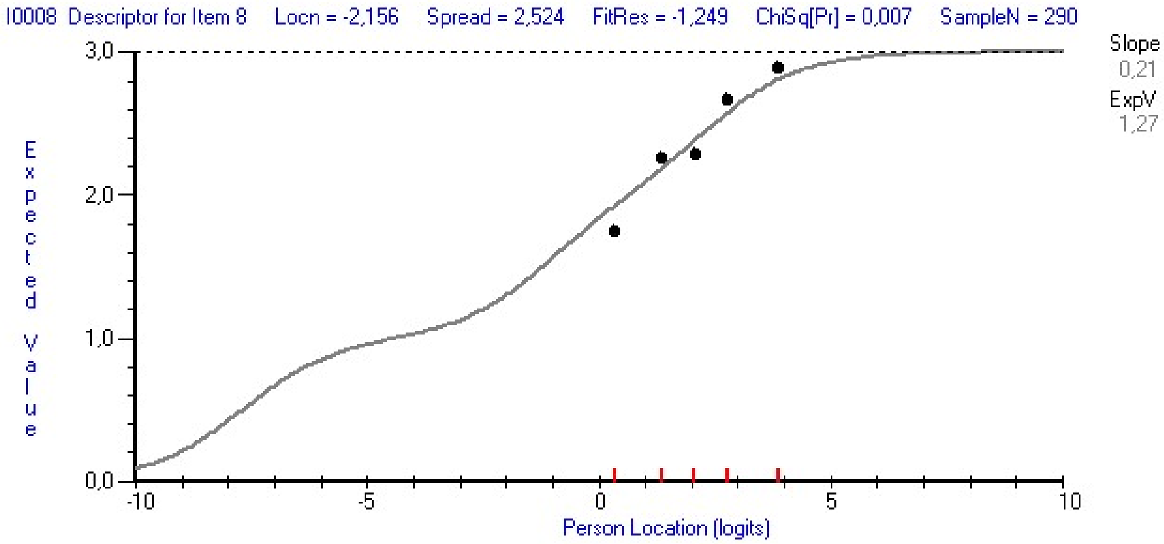

Figure S2. Item Characteristic Curve (ICC) for item 8.

References

1. Paakkari, O.; Torppa, M.; Kannas, L.; Paakkari, L. Subjective health literacy: Development of a brief instrument for school-aged children. *Scand. J. Public Health* **2016**, *44*, 751–757.
2. Bonde, A.H.; Stjernqvist, N.W.; Klinker, C.D.; Maindal, H.T.; Paakkari, O.; Elsborg, P. Translation and Validation of a Brief Health Literacy Instrument for School-Age Children in a Danish Context. *Health Lit. Res. Pract.* **2022**, *6*, e26–e29.
